# Comparison of multiple synthetic chondroinductive factors in pellet culture against a TGF-β positive control

**DOI:** 10.1016/j.ocarto.2023.100369

**Published:** 2023-05-12

**Authors:** Boushra Ajeeb, Michael Detamore

**Affiliations:** Stephenson School of Biomedical Engineering, University of Oklahoma, Norman, OK, 73019, USA

**Keywords:** Cartilage regeneration, Chondroinductive peptides, Chondrogenesis

## Abstract

Despite the advances in surgical and cell therapy regenerative techniques for cartilage repair, the challenge is to overcome an inferior fibrocartilage repair tissue*. In vitro*, TGF-β1 and TGF-β3 are the primary growth factors employed to induce chondrogenic differentiation. However, the clinical application of native proteins may present challenges regarding stability, cost, or reproducibility. Therefore, there remains an unmet clinical need for the identification of small chondroinductive synthetic molecules. From the literature, two peptides—CM10 and CK2.1—appear to be promising candidates; however, they have not been directly compared to TGF-β with human bone marrow-derived stem cells (hBMSCs). Similarly, two promising compounds—kartogenin and SM04690—have been reported in the literature to exhibit chondroinductive potential *in vivo* and *in vitro*; however, kartogenin was not directly compared against TGF-β. In the current study, we evaluated the chondroinductive potential of CM10, CK2.1, kartogenin, and SM04690, and directly compared them to each other and to a TGF-β3 positive control. Following 21 days of culture, none of the evaluated chondrogenic factors, either individually or even in combinations of two, resulted in a higher gene expression of chondrogenic markers as compared to TGF-β3. Additionally, no collagen II gene expression was detected except in the TGF-β3 positive control group. Given that the evaluated factors have confirmed efficacy in the literature, but not in the current study with a positive control, there may be value in the future identification of new chondroinductive factors that are less situation-dependent, with rigorous evaluations of their effect on chondrogenesis using positive controls.

## 1 Introduction

The complete regeneration of functional articular cartilage following traumatic or arthritic lesions is unlikely to occur owing to the low regenerative potential of cartilage. Cartilage injuries are common, especially in athletes where prevalence is around 37% [1], and it is currently estimated that 1 in 7 adults in USA suffer from osteoarthritis [2].

Treatment of cartilage lesions is challenging, and a variety of surgical techniques are available, of which bone marrow stimulation is the most commonly performed procedure [3]. Other procedures include autologous osteochondral transfer, osteochondral allograft transplantation [4,5], and matrix-assisted chondrocyte implantation autologous chondrocyte implantation (MACI) [6]. However, the outcome from currently available procedures is often an inferior fibrocartilage tissue that may lack the mechanical and functional properties of healthy hyaline cartilage.

Recent advances in cartilage regeneration focus on three main directions. The first is the development of off-the-shelf biomaterials that would fit into defects. The second direction is cell therapy, in which recent research focuses on using autologous or allogenic mesenchymal stem cells (MSCs) or chondrocytes or a combination of the two cell types to enhance cartilage regeneration. A third direction is focused on the search for chondroinductive factors, such as peptides or small compounds, to replace the current use of growth factors to induce the chondrogenic differentiation of MSCs and to regenerate cartilage.

The development of chondroinductive factors is an added value to both the biomaterials and cell therapy approaches. Peptides, for example, may be used to functionalize biomaterials to enhance their bioactivity [7]; additionally, chondroinductive factors may be added during cell therapy, or cells may be treated with chondroinductive factors prior to implantation to increase the likelihood of obtaining a repair tissue that resembles hyaline cartilage.

Several papers have reported the identification of peptides or small compounds with chondrogenic potential; however, comparison to a TGF-β3 positive control was lacking in most cases [8]. Additionally, different cell sources and culture methods have been used to evaluate different chondroinductive factors, rendering it challenging to compare the potency of different factors.

We recently presented an overview of peptides involved in cartilage regeneration [8], where we summarized promising peptides that have been evaluated both *in vitro* and *in vivo* for their chondroinductive potential. Among these peptides, we identified two leading candidates from the literature, CM10 and CK2.1, to evaluate and compare their chondrogenic potential with TGF-β3 using hBMSCs. CM10 (LIANAK) is a TGF-β1-simulating peptide that has been mainly evaluated for its wound healing potential [9] and collagen I synthesis, where it exhibited activity in the nanomolar and micromolar concentration range. Evaluation of CM10 as a chondroinductive peptide was first done by Renner and Liu in 2013 [10] as a soluble peptide; however, no significant activity was observed. In subsequent publications, CM10 was evaluated while conjugated to microspheres [11] or hydrogels [12], with the exception of one study [13] that evaluated soluble CM10 at a concentration of 100 ​μM with human periodontal ligament stem cells (hPLSCs), which found that a higher gene expression was observed for SOX-9, aggrecan, and collagen II at comparable values to a TGF-β positive control group.

CK2.1 is a peptide designed to block the interaction between the protein casein kinase 2 (CK2) and bone morphogenetic protein receptor type Ia (BMPRIa) inside the cell, thus leading to the activation of the BMP signaling pathway without the presence of a ligand. CK2.1 exhibited chondrogenic potential with C3H10T1/2 ​cells (i.e., a mouse cell line derived from C3H mouse embryos) following treatment with BMP-2. However, the *in vitro* evaluation with hBMSCs and comparison with TGF-β3 is yet to be done. Additionally, through a literature review of chondroinductive small compounds (unpublished), we identified two leading small compounds, SM04690 [14] and kartogenin [15], which have been reported to induce cartilage regeneration.

Kartogenin (KGN) was first reported in 2012 by Johnson et al. [15] as a heterocyclic drug-like molecule that induced the chondrogenic differentiation of hBMSCs; however, it was compared against dimethyl sulfoxide (DMSO) with no positive control. Kang et al. [16] enhanced the delivery of KGN by conjugating it to chitosan particles and found that conjugated KGN induced chondrogenic differentiation of hBMSCs more effectively than unconjugated kartogenin; however, there was no positive control from the TGF-β family included in the study. Liu et al. [17] evaluated lubricin and glycosaminoglycan (GAG) accumulation by rat BMSCs following treatment with TGF-β1, BMP-7, and/or KGN in monolayer culture. While KGN induced a significant increase in lubricin production, only the combination of TGF-β1, BMP-7, and KGN resulted in a significant increase in GAG content as compared to the untreated control group. Liu et al. [17] additionally evaluated the chondrogenic differentiation of rat BMSC/chondrocyte pellet co-cultures at different ratios in combination with KGN; however, KGN was added to all combinations, and no positive control was included *in vitro.*

SM04690 was identified as a Wnt signaling pathway inhibitor by Deshmukh et al. [14] using high throughput screening. SM04690 is currently in phase 3 clinical trials as a disease-modifying osteoarthritic drug (DMOAD) [18]. *In vitro*, SM04690 exhibited promising chondrogenic potential with hBMSCs, comparable to a TGF-β3 positive control [14].

In the current study, we evaluated and compared the chondrogenic potential of KGN, SM04690, CM10, and CK2.1, alongside a TGF-β3 positive control using hBMSCs following 21 days in pellet culture. Several studies in the literature suggest that an improvement in the chondrogenic differentiation of BMSCs may be observed under hypoxic conditions [[19], [20], [21]]; therefore, following preliminary studies that confirmed enhanced collagen II gene expression in hypoxia, we elected to perform the current study in 5% hypoxia.

Given that previous studies have reported synergistic effects between growth factors to enhance chondrogenic differentiation [[22], [23], [24], [25]], we additionally evaluated synergistic effects between small compounds and peptides. Finally, knowing that chondrogenesis may be donor-dependent [26], we evaluated the chondrogenic potential of select group concentrations with three different hBMSCs donors. Our hypothesis was that a combination of peptides and/or compounds would induce the chondrogenic differentiation of hBMSCs at levels equivalent or superior to TGF-β3.

## 2 Materials and methods

### 2.1 Cell culture

#### 2.1.1 Cell culture

Human BMSCs (hBMSCs) were purchased from RoosterBio, Inc., Frederick, MD, cultured in RoosterBasal™-MSC (Cat# SU-022, RoosterBio Inc., Frederick, MD) and supplemented with 20% RoosterBooster™-MSC (Cat# SU-003, RoosterBio Inc., Frederick, MD) and 1% penicillin/streptomycin (P/S, Cat# 15140122, ThermoFisher Scientific, Waltham, MA). The donor was a 23-year-old male. Cells were seeded at 3300 ​cells/cm^2^ and no medium exchange was required as per RoosterBio guidelines. Cells were passaged at 80–90% confluency and used at passage 3. For the multiple donors study, donor 1 was a 20-year-old female, donor 2 was a 19-year-old male, and donor 3 was a 25-year-old male.

#### 2.1.2 Pellet formation

For chondrogenic differentiation, 100,000 ​cells were added to the wells of U-bottom 96-well plates (Cat# 10861-564, VWR, Radnor, PA) following treatment with Anti-Adherence Rinsing Solution (StemCell Technologies cat# 07010). The plates were centrifuged at 100×*g* for 3 ​min for pellet formation.

#### 2.1.3 Differentiation medium

Negative control medium was prepared using DMEM/high glucose/GlutaMAX™ (Cat# 10566016, ThermoFisher Scientific, Waltham, MA), with 1% P/S, insulin, human transferrin and selenous acid (ITS)+ premix 1*x* (Cat# 354352, Corning, Corning, NY), sodium pyruvate 1 ​mM (Cat# 11360070, ThermoFisher Scientific, Waltham, MA), MEM non-essential amino acids 1*x* (Cat# 11140050, ThermoFisher Scientific, Waltham, MA), ascorbate-2-phosphate 50 ​μg/ml (Cat# A8960, Sigma-Aldrich, St. Louis, MO), and dexamethasone 100 ​nM (Cat# D4902, Sigma-Aldrich, St. Louis, MO). For the positive control, TGF-β3 10 ​ng/ml (Cat# 8420-B3-005, R&D biosystems, Minneapolis, MN) was added to the negative control medium. For experimental groups, KGN (Cat# HY-16268, MedChemExpress, Monmouth Junction, NJ), SM04690 (Cat# HY-109049, MedChemExpress, Monmouth Junction, NJ), CM10 (Sequence LIANAK, Genscript, New Jersey), and/or CK2.1 (Sequence: QIKIWFQNRRK-WKKMVPSDPSYEDMGGC, Genscript, New Jersey) were added to the control medium at concentrations that were based on the literature and summarized in Table 1. Cells were incubated at 37°C in hypoxia (5% O_2_) for 21 days.

**Table 1:** List of experimental groups and concentrations evaluated.

Experimental groups	Concentrations
Kartogenin (KGN)	1 ​μM, 10 ​μM
SM04690 (SM)	30 ​nM, 100 ​nM
CM10	100 ​μM, 200 ​μM
CK2.1	100 ​nM, 500 ​nM
Combinations	
CM10/CK2.1	100 ​μM/100 ​nM; 100 ​μM/500 ​nM 200 ​μM/100 ​nM; 200 ​μM/500 ​nM
CM10/KGN	100 ​μM/1 ​μM; 100 ​μM/10 ​μM
CM10/SM04690	100 ​μM/30 ​nM; 100 ​μM/100 ​nM
CK2.1/KGN	100 ​nM/1 ​μM; 100 ​nM/10 ​μM
CK2.1/SM04690	100 ​nM/30 ​nM; 100 ​nM/100 ​nM

### 2.2 Real-time quantitative polymerase chain reaction

Total RNA was isolated from the pellets on day 21 using Quick-RNA Miniprep Plus Kit (Zymo Research, cat# R1058) following the kit handbook. Briefly, pellets were stored in DNA/RNA shield, then digested with Proteinase K and digestion buffer for 4 ​h at room temperature, followed by the addition of an equal volume of lysis buffer. DNA transcription was done using the High-Capacity cDNA Reverse Transcription kit (ThermoFisher Scientific, cat# 4368813). Real-time quantitative polymerase chain reaction (RT-qPCR) was performed using a qTOWER (AnalytikJena, Jena, Germany) and TaqMan® Fast Universal PCR Master Mix (ThermoFisher Scientific, cat# 4366073). Preconfigured Taqman probes were used and are listed in Table 2. Eight samples from each group (n ​= ​8) were tested in duplicate. Relative levels of gene expression were calculated using the comparative ΔΔCt method for relative quantification. The negative control group was the calibrator group and GAPDH was used as the endogenous reference gene.

**Table 2:** TaqMan probes information.

Target gene	Assay ID	Interrogated sequence	Probe context sequence
GAPDH	Hs02786624_g1	NM_001256799.2	CGCTGCCAAGGCTGTGGGCAAGGTC
ACAN	Hs00153936_m1	NM_001135.3	CCGCTGCCAGGGATCCTTCCTACTT
Collagen I	Hs00164004_m1	NM_000088.3	AAGACGAAGACATCCCACCAATCAC
Collagen II	Hs00264051_m1	NM_001844.4	TGGTCTTGGTGGAAACTTTGCTGCC
SOX-9	Hs00165814_m1	NM_000346.3	GAGCACTCGGGGCAATCCCAGGGCC

### 2.3 Statistical analysis

All graphs and statistical analyses were performed using GraphPad Prism 9 (GraphPad Software Inc. La Jolla, CA). One-way ANOVA analyses were performed followed by a Tukey post hoc correction. Results were considered significant at p ​< ​0.05. Showing ∗∗∗∗p ​< ​0.0001, ∗∗∗p ​< ​0.001, ∗∗p ​< ​0.01, and ∗p ​< ​0.05. Error bars on graphs show the standard error of the mean.

## 3 Results

### 3.1 Pellet formation

Pellets successfully formed in all groups within 24 ​h of culture. Over 21 days of culture, the pellets maintained their form in all groups.

### 3.2 Gene expression

**Fig. 1:**
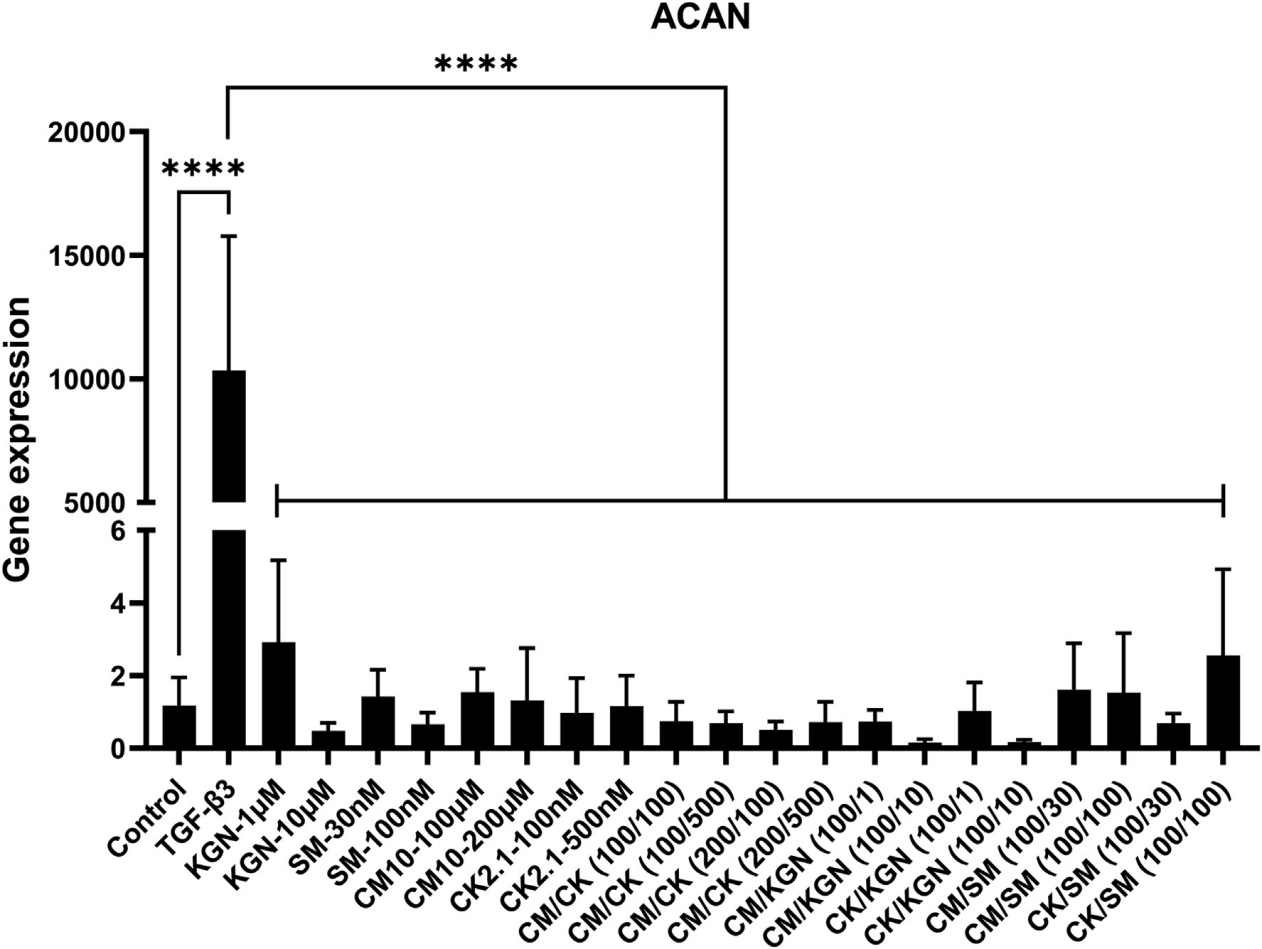
Chondrogenic gene expression of ACAN in hBMSCs spheroids after 21 days in culture. No statistically significant differences were observed between groups except for TGF-β3, which had significantly higher aggrecan expression compared to all other groups, including a 10,000-fold larger value compared to the negative control. ∗∗∗∗Refers to significant difference p ​< ​0.0001, n ​= ​6–8. Reported values are mean ​+ ​standard deviation. SM ​= ​SM04690, CK ​= ​CK2.1, CM ​= ​CM10, CM/CK (100/100) ​= ​CM10 at 100 ​μM and CK2.1 and 100 ​nM, CM/CK(100/500) ​= ​CM10 at 100 ​μM and CK2.1 and 500 ​nM, CM/CK (200/100) ​= ​CM10 at 200 ​μM and CK2.1 and 100 ​nM, CM/CK(200/500) ​= ​CM10 at 200 ​μM and CK2.1 and 500 ​nM, CM/KGN (100/1) ​= ​CM10 at 100 ​μM and kartogenin at 1 ​μM, CM/KGN (100/10) ​= ​CM10 at 100 ​μM and kartogenin at 10 ​μM, CK/KGN (100/1) ​= ​CK2.1 ​at 100 ​nM and kartogenin at 1 ​ ​μM, CK/KGN (100/10) ​= ​CK2.1 ​at 100 ​nM and kartogenin at 10 ​μM, CM/SM (100/30) ​= ​CM10 at 100 ​μM and SM04690 at 30 ​nM, CM/SM (100/100) ​= ​CM10 at 100 ​μM and SM04690 at 100 ​nM, CK/SM (100/30) ​= ​CK2.1 at 100 ​nM and SM04690 at 30 ​nM, CK/SM (100/100) ​= ​CK2.1 at 100 ​nM and SM04690 at 100 ​nM.

**Fig. 2:**
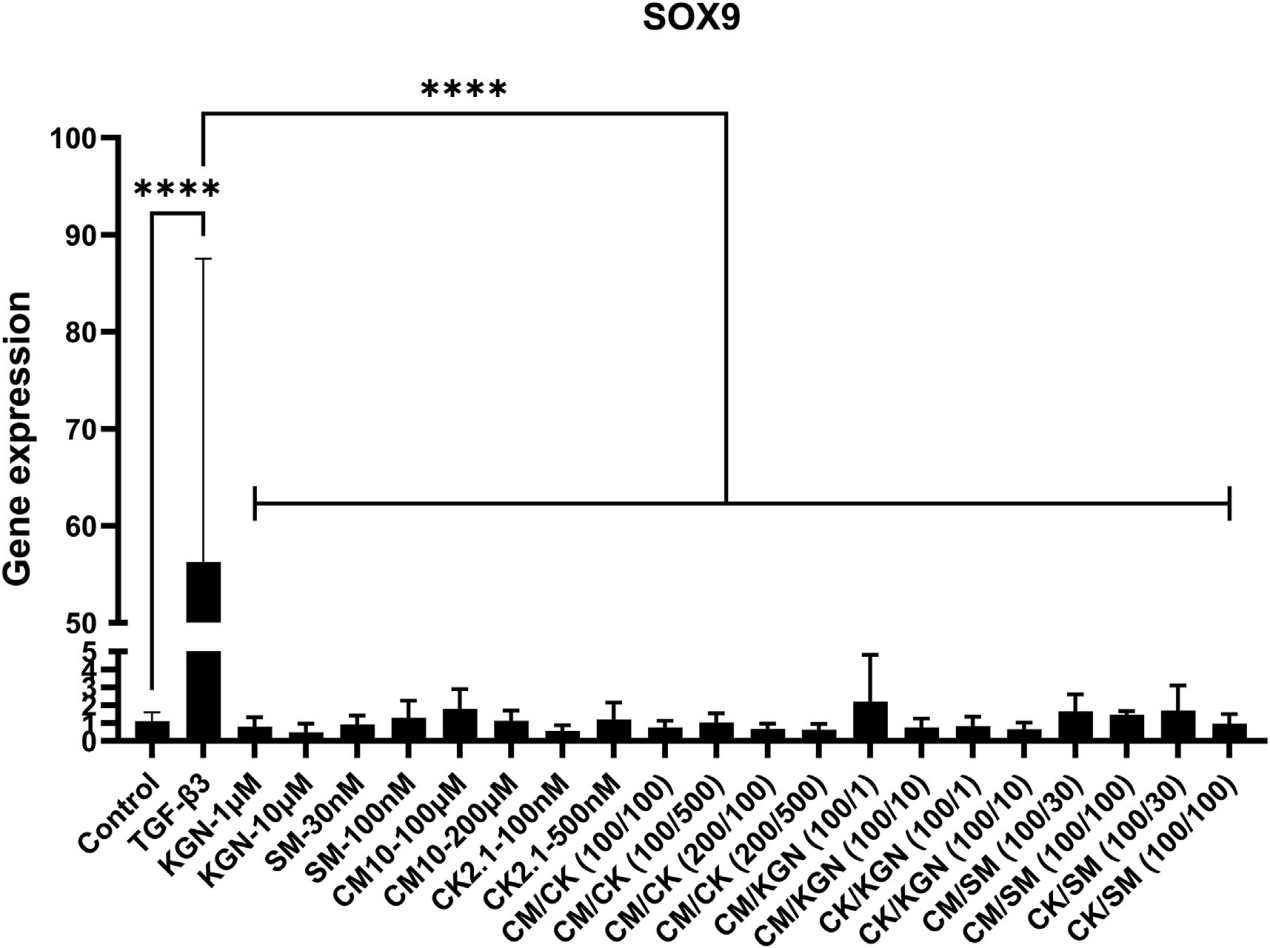
Chondrogenic gene expression of SOX-9 in hBMSCs spheroids after 21 days in culture. No significance was observed between groups except for TGF-β3 with a 50-fold higher gene expression as compared to the negative control. ∗∗∗∗Refers to significant difference p ​< ​0.0001, n ​= ​6–8. Reported values are mean ​+ ​standard deviation. SM ​= ​SM04690, CK ​= ​CK2.1, CM ​= ​CM10, CM/CK (100/100) ​= ​CM10 at 100 ​μM and CK2.1 and 100 ​nM, CM/CK(100/500) ​= ​CM10 at 100 ​μM and CK2.1 and 500 ​nM, CM/CK (200/100) ​= ​CM10 at 200 ​μM and CK2.1 and 100 ​nM, CM/CK(200/500) ​= ​CM10 at 200 ​μM and CK2.1 and 500 ​nM, CM/KGN (100/1) ​= ​CM10 at 100 ​μM and kartogenin at 1 ​μM, CM/KGN (100/10) ​= ​CM10 at 100 ​μM and kartogenin at 10 ​μM, CK/KGN (100/1) ​= ​CK2.1 at 100 ​nM and kartogenin at 1 ​ ​μM, CK/KGN (100/10) ​= ​CK2.1 at 100 ​nM and kartogenin at 10 ​μM, CM/SM (100/30) ​= ​CM10 at 100 ​μM and SM04690 at 30 ​nM, CM/SM (100/100) ​= ​CM10 at 100 ​μM and SM04690 at 100 ​nM, CK/SM (100/30) ​= ​CK2.1 at 100 ​nM and SM04690 at 30 ​nM, CK/SM (100/100) ​= ​CK2.1 at 100 ​nM and SM04690 at 100 ​nM.

For ACAN, no significant differences were observed among groups except for the TGF-β3 positive control group, which was significantly larger than all other groups, including a 10,000-fold (p ​< ​0.0001) larger ACAN gene expression compared to the negative control (Fig. 1). Regarding SOX-9, TGF-β3 induced a 50-fold (p ​< ​0.0001) higher gene expression compared to the negative control (Fig. 2), whereas no significant differences were observed among the remaining experimental groups and the negative control. Surprisingly, collagen II expression was only detected in the TGF-β3 positive control; therefore, relative gene expression could not be calculated for this single group.

**Fig. 3:**
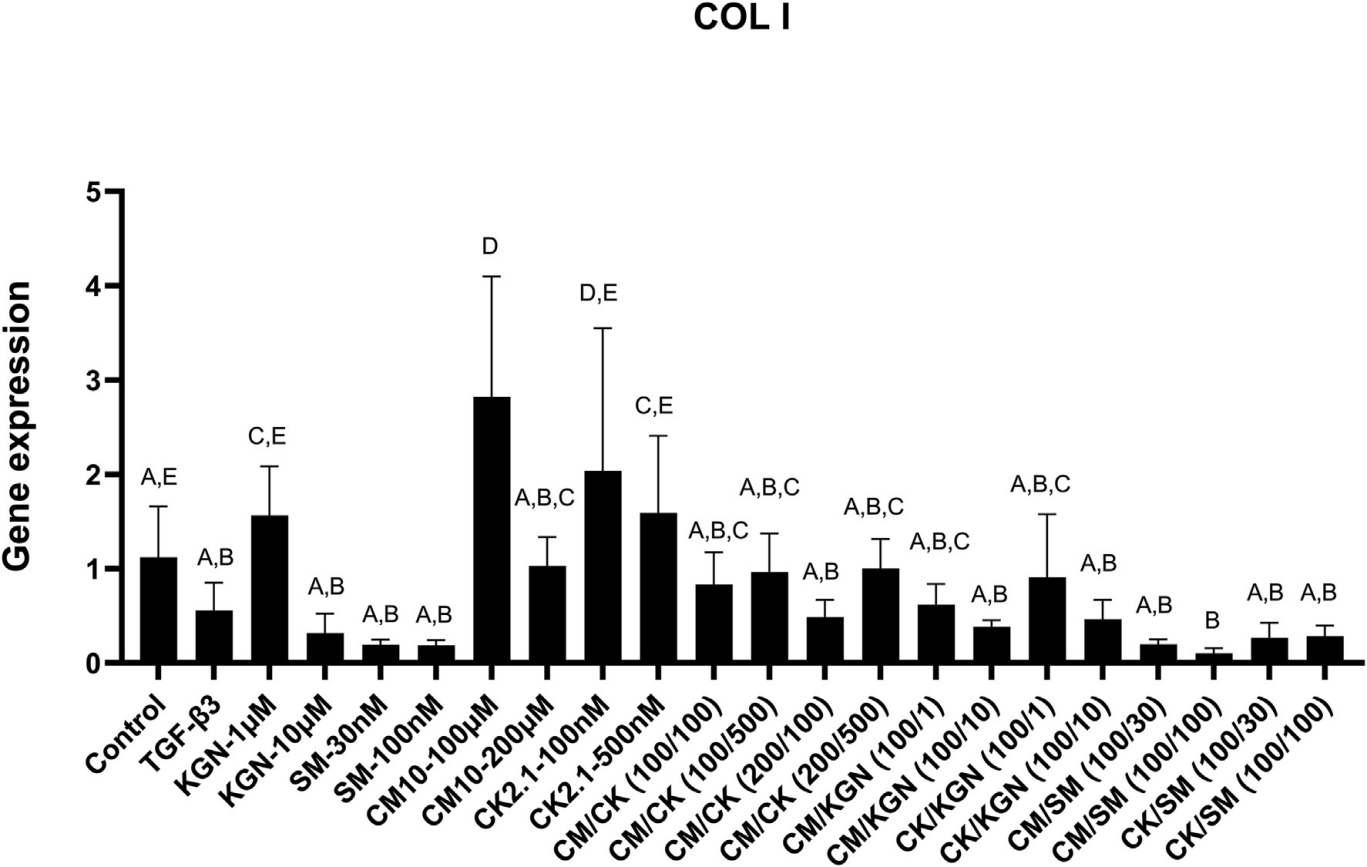
Gene expression of collagen I of hBMSCs spheroids after 21 days in culture. A variation in the gene expression of collagen I by hBMSCs was observed in response to the evaluated compounds and peptides. CM10 (100 ​μM) had the highest gene expression with 2.5-fold higher expression compared to the control group. CK2.1 exhibited higher gene expression of collagen I by 1.8 folds and 1.4 folds at 100 ​nM and 500 ​nM, respectively. The combination of compounds and peptides did not induce a significant difference in collagen I gene expression as compared to the negative control group. Groups with the same letters indicate no significance, n ​= ​6–8. Reported values are mean ​+ ​standard deviation. SM ​= ​SM04690, CK ​= ​CK2.1, CM ​= ​CM10, CM/CK (100/100) ​= ​CM10 at 100 ​μM and CK2.1 and 100 ​nM, CM/CK(100/500) ​= ​CM10 at 100 ​μM and CK2.1 and 500 ​nM, CM/CK (200/100) ​= ​CM10 at 200 ​μM and CK2.1 and 100 ​nM, CM/CK(200/500) ​= ​CM10 at 200 ​μM and CK2.1 and 500 ​nM, CM/KGN (100/1) ​= ​CM10 at 100 ​μM and kartogenin at 1 ​μM, CM/KGN (100/10) ​= ​CM10 at 100 ​μM and kartogenin at 10 ​μM, CK/KGN (100/1) ​= ​CK2.1 at 100 ​nM and kartogenin at 1 ​ ​μM, CK/KGN (100/10) ​= ​CK2.1 at 100 ​nM and kartogenin at 10 ​μM, CM/SM (100/30) ​= ​CM10 at 100 ​μM and SM04690 at 30 ​nM, CM/SM (100/100) ​= ​CM10 at 100 ​μM and SM04690 at 100 ​nM, CK/SM (100/30) ​= ​CK2.1 at 100 ​nM and SM04690 at 30 ​nM, CK/SM (100/100) ​= ​CK2.1 at 100 ​nM and SM04690 at 100 ​nM.

Interestingly, no significant difference was observed in the collagen I gene expression between the negative control and the TGF-β3 positive control (Fig. 3); however, a variation was observed in response to the evaluated compounds and peptides. CM10 (100 ​μM) had the highest gene expression with a 2.5-fold (p ​< ​0.0001) higher expression compared to the negative control group. CK2.1 had a collagen I gene expression that was 40% (p ​= ​0.0978) and 50% (p ​= ​0.9782) higher at 100 ​nM and 500 ​nM than the negative control, respectively, although these differences were not significant. The combination of compounds and peptides did not induce a significant difference in collagen I gene expression as compared to the negative control group.

**Fig. 4:**
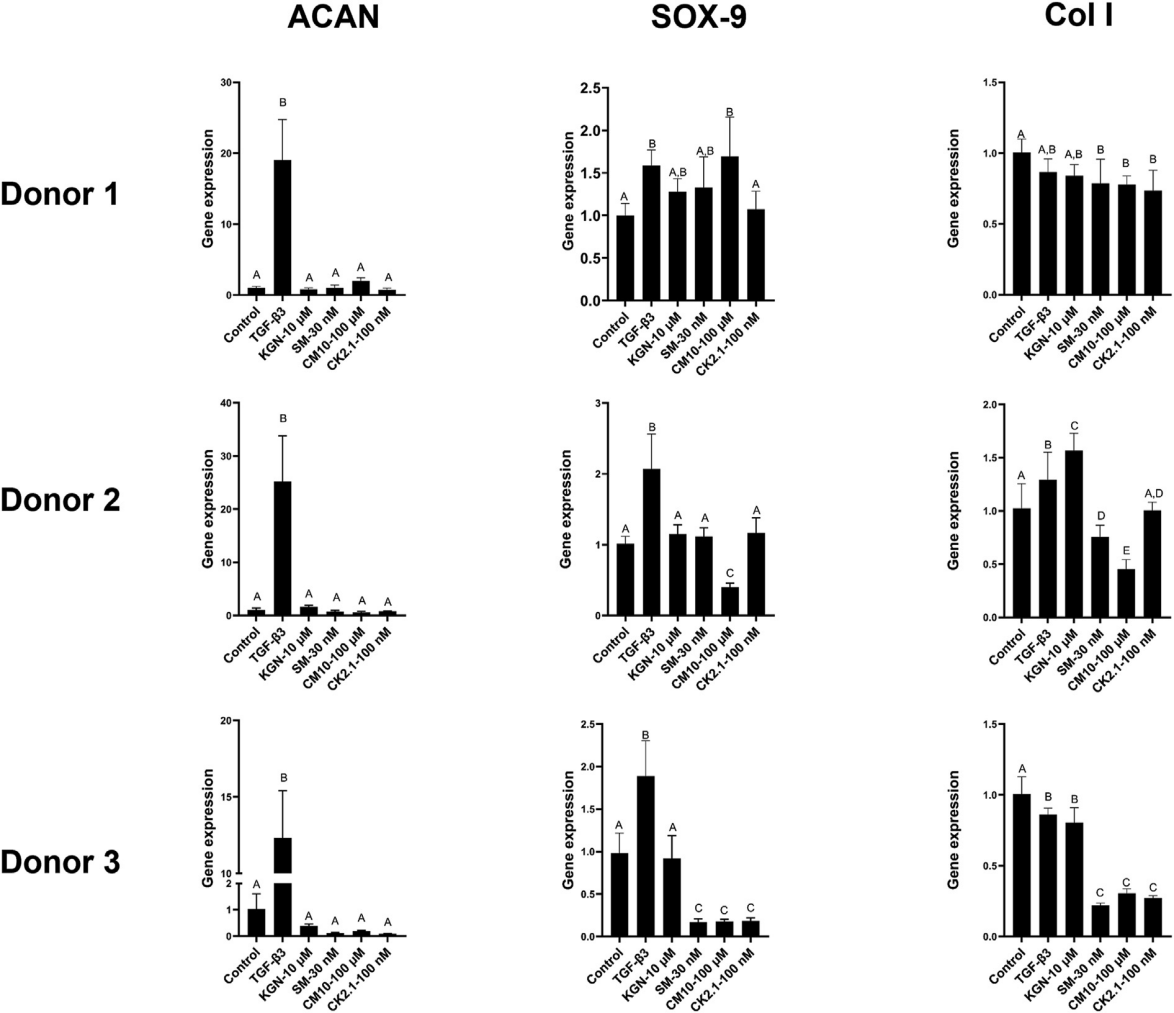
Gene expression of ACAN, SOX-9, and collagen I of hBMSCs spheroids from 3 different donors after 21 days in culture. For ACAN, no statistically significant differences were observed between groups except for TGF-β3 with all donors. Donor-based variation in the gene expression of SOX-9 in response to experimental groups was observed. Notably, CM10 had a significantly higher gene expression of SOX-9 as compared to the negative control only with donor 1 (Female, 20 ​yrs). SM04690, CM10, and CK2.1 induced a significantly lower gene expression of collagen I as compared to the negative control with all donors, whereas KGN had variable outcomes based on the donor. Groups with the same letters indicate no significance, n ​= ​6–8. Reported values are mean ​+ ​standard deviation. SM ​= ​SM04690, CK ​= ​CK2.1, CM ​= ​CM10.

For the multiple donors study (Fig. 4), no significant differences in the gene expression of ACAN were observed among groups, except for TGF-β3 group across all donors, which is in agreement with the results obtained in the first study. For SOX-9, CM10 exhibited a higher gene expression as compared to the negative control in donor 1; however, with donor 2 and 3 the gene expression of SOX-9 in the CM10 group was significantly lower than the negative control. For KGN, no significant differences compared to the negative control were observed with all 3 donors. As for SM04690 and CK2.1, no significant differences were observed compared to the negative control with donors 1 and 2, whereas a significantly lower SOX-9 gene expression was observed with donor 3. As for collagen II, and as observed in study 1, gene expression was only detected in the TGF-β3 positive control; therefore, relative gene expression could not be calculated.

## 4 Discussion

The current study was the first study to evaluate and compare the chondroinductive potential of multiple promising peptides and compounds with TGF-β3 as a positive control. Additionally, this is the first report that evaluates the combination of peptides and compounds on the chondrogenic differentiation of hBMSCs. Our hypothesis was not supported, as there were no individual factors or combinations of factors that outperformed TGF-β3 in chondrogenesis.

Interestingly, none of the evaluated KGN concentrations in the current study induced the gene expression of collagen II or enhanced ACAN or SOX-9 gene expression as compared to the negative or positive control following 21 days in spheroid culture. The absence of increased gene expression as compared to TGF-β3 is in agreement with what was observed by Music et al. [27] with hBMSC macropellet and micropellet cultures, which showed that KGN was inferior to TGF-β1 in terms of chondrogenic potential following 7 or 14 days of continuous KGN treatment. The first KGN paper by Johnson et al. [15] reported a higher gene expression of chondrogenic markers (ACAN and collagen II) on day 21 following 3 days of treatment with KGN at concentrations 100 ​nM, 1 ​μM, and 10 ​μM in hBMSCs pellet culture. While there was no TGF-β positive control in that study, it may be possible that continuous treatment with KGN for more than 3 days could hinder chondrogenesis; therefore, additional evaluation is warranted given that 3 days of TGF-β3 treatment is known to work as a chondrogenesis positive control. Interestingly, a 2020 abstract reported the synthesis of KA-34 [28], a more potent and stable KGN analog, and additionally a phase I clinical trial [29] has been completed with KA-34, but no publication is currently available. Hence, future work might include investigating the chondrogenic potential of KA-34.

SM04690 was reported as a promising DMOAD by Deshmukh et al. [14,30] and its chondroinductive potential was demonstrated with hBMSCs in pellet culture (150,000 ​cells/pellet) and in monolayer culture (40,000 ​cells/well in 48 well plate) at 30 ​nM as compared to treatment with 20 ​ng/ml of TGF-β3 with medium changes done every 5 days [14]. In the current study, SM04690-treated groups surprisingly exhibited no significant difference in the gene expression of ACAN and SOX-9 relative to the negative control, and collagen II gene expression was not observed. One possible explanation for the differences in outcomes between studies is that Deshmukh et al. [14] used incomplete chondrocyte differentiation medium (iCDM; Lonza) and chondrogenesis was evaluated in normoxia, whereas in the current study, DMEM medium with individually prepared supplements was used and cells were cultured in hypoxia.

The current study was the first study to evaluate the chondrogenic potential of CM10 with hBMSCs as compared to a positive control. No significant difference in the gene expression of ACAN or SOX-9 was observed with CM10 treatment as compared to the negative control. On the other hand, treatment with 100 ​μM CM10 resulted in the highest gene expression of collagen I as compared to the negative control group that might indicate why CM10 was successfully used in wound healing applications [9,31]. The chondrogenic potential of CM10 was first evaluated in 2013 with hBMSCs at 0.05 and 0.1 ​μM^10^; however, no promising outcome was observed. Another study [13] reported enhanced gene expression of chondrogenic markers following treatment with 100 ​μM of CM10; however, hPLSCs were the cell source, which may respond differently from hBMSCs. Interestingly, other studies reported positive outcomes with CM10-functionalized microspheres [11] and CM10-functionalized hydrogels [12]; however, no positive control was included in the first study. It could be that CM10 will exhibit a more prominent activity when presented on a scaffold versus as a soluble peptide, which requires additional investigation in the future.

The current study is the first report to evaluate the chondrogenic potential of CK2.1 with hBMSCs in comparison to TGF-β3. CK2.1 showed promising outcomes in the original publication [32]; however, the peptide was evaluated with C3H10T1/2 ​cells, which are a mouse cell line that are functionally similar to mesenchymal stem cells and undergo chondrogenesis when induced by cellular condensation and bone morphogenetic protein-2 (BMP-2) [33]. The current study used the same concentrations reported in the original paper (100 ​nM and 500 ​nM); however, no significant difference in the gene expression of ACAN and SOX-9 chondrogenic markers relative to the negative control were observed. Additionally, the gene expression of collagen II was not observed. In addition to the difference in cell source, the original paper used DMEM medium with 10% fetal bovine serum (FBS) with no additional supplements, whereas the current study used a serum-free medium.

Combinations between growth factors have been shown to enhance the chondrogenic differentiation of MSCs [[22], [23], [24], [25]]. The current study evaluated different combinations between CM10, CK2.1, KGN, and SM04690; however, none of the combinations resulted in a higher gene expression of chondrogenic markers as compared to the negative control.

To eliminate the effect of donor variation on the results obtained in the first study, we evaluated the chondroinductive potential of select group concentrations: CM10 (100 ​μM), CK2.1 (100 ​nM), KGN (10 ​μM), and SM04690 (30 ​nM), with 3 different donors. While we did observe donor-dependent variation in the gene expression of SOX-9 among experimental groups; however, and as seen with the first study, the lack of collagen II expression and absence of higher ACAN gene expression indicated that there was no evidence of chondrogenesis with any of the evaluated peptides and compounds.

Given that prior studies have demonstrated chondroinduction with CM10, CK2.1, KGN, and SM04690, and used a variety of different conditions as delineated above, we cannot conclude that these factors are not chondroinductive. Instead, we conclude that evidence of chondroinduction was not observed in the conditions employed in the current study, and therefore that *in vitro* chondroinduction with these factors may depend on the cell source and culture conditions (e.g., medium composition, monolayer vs. pellet vs. 3D scaffold, hypoxia vs. normoxia). Additionally, the evaluated factors could be chondroinductive if applied transiently [[34], [35], [36], [37]] or have alternate modes of action such as stabilizing chondrogenesis if applied beyond day 7 or 14 of chondrogenesis, or could be fibrogenic rather than chondrogenic, which entreats additional investigation in the future. Furthermore, once we move *in vivo*, there are other parameters such as the immune system and/or mechanical stimulation that may affect the potency of chondroinductive factors.

TGF-β3 is the main growth factor employed for chondrogenesis, as it is known to induce chondrogenesis with different cell sources and culture conditions, including in the current study. Therefore, the current study underscores the importance of identifying factors (as alternatives to TGF-β3) that are not situation dependent, and identification of new factors might be the key to enhancing the outcome of biomaterial-based or cell therapy-based cartilage regeneration techniques. In future studies that do identify new factors for chondroinduction, comparing to other published factors is recommended if possible, and including a positive control is strongly recommended so that there is context for the extent of observed chondroinduction. Furthermore, as BMSCs tend to acquire hypertrophic properties during chondrogenic induction, it is imperative that hypertrophy markers (e.g., collagen X) be evaluated in future studies to clearly highlight the difference between various chondrogenic compounds.

## Author contributions

M.S. Detamore contributed to the vision and conception of the paper, B. Ajeeb drafted the paper and performed the data collection and analysis.

## Funding

We gratefully acknowledge support from the Stephenson Graduate Fellowship (to Boushra Ajeeb). We acknowledge funding from the 10.13039/100000069National Institute of Arthritis and Musculoskeletal and Skin Diseases of the 10.13039/100000002National Institutes of Health (R21 AR077800). We acknowledge financial support provided by the University of Oklahoma Libraries’ Open Access Fund.

## Declaration of competing interest

No competing financial interests exist.
